# META-ANALYSES OF THE EFFECTS OF EXERCISE ON ALZHEIMER’S DISEASE: EVALUATING SYNTHESIS REDUNDANCY AND RESEARCH WASTE

**DOI:** 10.1093/geroni/igae098.0298

**Published:** 2024-12-31

**Authors:** Mahederemariam Dagne

**Affiliations:** Marymount University, Arlington, Virginia, United States

## Abstract

Research redundancy emerges when studies are unnecessarily duplicated, which not only depletes resources but also inflates research waste. In the last twenty years, we have been seeing a significant redundancy in the number of systematic reviews and meta-analyses evaluating exercise and physical activity interventions for cognitive function in older adults with cognitive impairments, particularly those with Alzheimer’s disease. This presentation aims to dissect the prevalence of repeated studies within published meta-analyses through a living umbrella systematic review. It scrutinizes the overlap of randomized controlled trials (RCTs) across these analyses to elucidate the extent of redundancy and its implications on the accumulation of knowledge. Our analysis provides a critical assessment of the current landscape, charting a path toward more strategic, impactful research practices. By pinpointing specific instances of excessive duplication, we aim to foster a dialogue on refining research

